# A pilot trial of human amniotic fluid for the treatment of COVID-19

**DOI:** 10.1186/s13104-021-05443-9

**Published:** 2021-01-22

**Authors:** Craig H. Selzman, Joseph E. Tonna, Jan Pierce, Camila Vargas, Chloe Skidmore, Giavonni Lewis, Nathan D. Hatton, John D. Phillips

**Affiliations:** 1 Division of Cardiothoracic Surgery, University of Utah School of Medicine, 3C 127, 30 N 1900 E, Salt Lake City, UT 84132 USA; 2 Cell Therapy and Regenerative Medicine Program, University of Utah School of Medicine, Salt Lake City, UT USA; 3 Division of General Surgery, University of Utah School of Medicine, Salt Lake City, UT USA; 4 Pulmonary and Criticial Care Medicine, University of Utah School of Medicine, Salt Lake City, UT USA; 5 Division of Hematology, University of Utah School of Medicine, Salt Lake City, UT USA

**Keywords:** COVID-19, Amniotic fluid, Inflammation, C-reactive protein

## Abstract

**Objective:**

Vertical transmission from SARS CoV-2-infected women is uncommon and coronavirus has not been detected in amniotic fluid. Human amniotic products have a broad immune-mediating profile. Observing that many COVID-19 patients have a profound inflammatory response to the virus, we sought to determine the influence of human amniotic fluid (hAF) on hospitalized patients with COVID-19.

**Results:**

A 10-patient case series was IRB-approved to study the impact of hAF on hospitalized patients with documented COVID-19. Nine of the 10 patients survived to discharge, with one patient succumbing to the disease when enrolled on maximal ventilatory support and severe hypoxia. The study design was altered by the IRB such that the last 6 patients received higher dose of intravenous hAF. In this latter group, patients that had observed reductions in C-reactive protein were associated with improved clinical outcomes. No hAF-related adverse events were noted. Acknowledging some of the inherent limitations of this case series, these results inform and catalyze a larger scaled randomized prospective trial to further investigate hAF as a therapy for COVID-19.

*Trial Registration* ClinicalTrials.gov: NCT04319731; March 23, 2020

## Introduction

Early data in the COVID-19 pandemic suggested that vertical transmission from SARS CoV-2-infected women is uncommon and virus has not been detected in the amniotic fluid [1–4]. These observations raise the question of whether or not there is something intrinsically protective in the fluid. Indeed, human amniotic products have a broad immune mediating profile [5]. Human amniotic membrane (hAM) and human amniotic fluid (hAF) have been shown to reduce inflammation, have antimicrobial properties, and confer a low risk of immunogenicity [6–11]. Purified hAF is non-antigenic solution devoid of any cellular products (i.e., it is not to be confused with umbilical cord-derived, AF-derived stem cell products, or AF embolism) that nature developed [5, 12]. Hence, amniotic products make ideal biocompatible scaffolds for the treatment of diverse conditions, including but not limited to intractable epithelilal defects, burns, diabetic/peripheral vascular ulcers, partial limbal cell deficiencies, peripheral nerve regeneration, tendon repair, and Stevens-Johnson syndrome [13–15].

Our group has developed experience with the clinical use of amniotic products and is currently using hAM as a tissue allograft in burn patients, for digital ulcers in scleroderma patients, as a nerve wrap to protect nerves from adhesions post-surgery, and as a trachea-stenosis-tracheal stent covering. Additionally, our burn unit has successfully injected purified hAF into more than 500 burn patient wounds to augment graft survival. hAF is also currently being used experimentally under IND approval to treat ocular graft versus host disease and ocular PRK. Given the use of amniotic products for the treatment of inflammation in other conditions, our group proposed an experimental and innovative use of hAF as its impact on cardiopulmonary failure has not been previously investigated. In light of the profound local and systemic inflammation associated with COVID-19 [16], it is our global hypothesis that hAF could significantly mitigate the progression of disease. An expedited protocol was approved by the University of Utah Institutional Review Board to study the influence of hAF in 10 patients with confirmed COVID-19. Herein, we report the results of this case series.

## Main text

Purified hAF is a cell-free, non-antigenic solution (not to be confused with umbilical cord-derived or AF-derived stem cell products) that is processed and manufactured at the University of Utah for clinical use [8, 12]. The initial trial design identified two cohorts of hospitalized, symptomatic, and laboratory verified SARS CoV-2 patients: (1) mechanically ventilated patients were administered hAF both intravenously (3 cc) and nebulized (3 cc) for 5 days; and (2) non-mechanically ventilated patients were administered only nebulized (3 cc) hAF. After the first 4 patients, concerns with utilizing aerosolized therapies in COVID-19 patients required a temporary hold and subsequent re-design. Thereafter (the last 6 patients), all received a higher dose of intravenous hAF (10 cc) for 5 consecutive days. Patients were eligible for this study if there were over the age of 18, had a SARS CoV-2 laboratory positive test obtained within 14 days of enrollment, were hospitalized, and symptomatic (e.g., cough, fevers, shortness of breath, sputum production). There were no exclusion criteria for the case series. Furthermore, to the best of our knowledge, there are no absolute contraindication for the clinical use of our purified hAF. Participants were enrolled over the span of seven weeks between late March and early May of 2020.

The demographics (Table 1) of the total 10 patient cohort included the following: 40% female; average age of 51.9 years old (range 24–76); five White, two American Indian/Alaska Native, two Hispanic, and one as unknown/other. Average weight was 91 kg (range 42–140). Eight of the 10 participants had underlying comorbid conditions, of which the most common were diabetes (n = 6) and hypertension (n = 5). As this is a small case series, pertinent information is provided in a patient-by-patient manner (Table 1). Of the 10 patients enrolled, seven were admitted directly to the intensive care unit (ICU). All ten patients required oxygen support during their hospital admission. Three patients required a nasal cannula or simple mask as their highest level of support, while two were placed on a high flow nasal cannula as their highest support. Five patients required mechanical ventilation. One patient (#1) was enrolled on high-level ventilatory support (100% FiO2, 18 mmHg PEEP) in the prone position and ultimately required 41 days of veno-veno extracorporeal membrane oxygenation (initiated after 5^th^ day of hAF treatment) as well as tracheostomy, which was removed prior to discharge. Another patient (#6) required a trachestomy and was discharged with this still in place, although he was no longer on mechanical ventilation. Of the nine patients who have been discharged alive, 7 required home oxygen use.

**Table 1:** Demographics and medical history

Variable	n
Age (mean, range)	52, 24–76
Female sex	4
Weight (kg; mean, range)	91 (42–140)
Race/Ethnicity	
*White*	5
*American Indian/Alaskan Native*	2
*Hispanic*	2
*Unknown/other*	1
Medical History	
*Anemia*	1
*Coronary artery disease*	1
*Diabetes*	6
*Elevated liver enzymes*	1
*Heart murmur*	1
*Hyperlipidemia*	2
*Hypertension*	5
*Liver disease*	1
*Mitral valve disease*	1
*Respiratory disease (e.g., asthma)*	2
*No past medical history*	2

Results of primary outcome measures included hospital length of stay, ICU length of stay, ventilator-free days, and supplemental oxygen needs at the time of discharge (Table 2). The 4 patients who were mechanically ventilated and ultimately discharged alive average 13.5 ventilator-free days (range 5–28). With regard to supplemental oxygen use at the time of discharge, 2 patients were entirely on room air, 6 were requiring continuous oxygen, and 1 required intermittent oxygen use. Average hospital length of stay (n = 10) was 20.4 days (range 5–56 days). Nine of 10 patients were admitted to the ICU at some point during their hospitalization. Average ICU length of stay for surviving patients (n = 8) was 17.38 days (range 2–56 days). For this subgroup of 8 patients, the average ICU-free days/length of stay on the medical floor was 5.9 days (range 1–19 days).

**Table 2:** Outcomes of 10 enrolled patients

Patient	Admit	Initial O2	Maximum O2	Co-Rx	CRP	IL-6	D-dimer	LDH	Discharge
1	ICU	ETT	ECMO/Trach	HCQ,	+ 30%	+ 38%	+ 138%	− 27%	Alive
2	Floor	NC	NC	HCQ	N/A	N/A	N/A	− 28%	Alive
3	Floor	NC	ETT	HCQ, Rem	N/A	N/A	N/A	− 20%	Deceased
4	ICU	NC	NC	HCQ	N/A	N/A	N/A	N/A	Alive
5	ICU	ETT	ETT	HCQ	− 84%	− 60%	− 21%	− 22%	Alive
6	ICU	ETT	Trach	HCQ-AZ	− 91%	0	− 42%	N/A	Alive
7	ICU	HFNC	HFNC	HCQ	+ 46%	+ 109%	+ 818%	+ 109%	Alive
8	ICU	NC	HFNC	AZ	− 87%	0	0	− 21%	Alive
9	ICU	NC	Simple mask	HCQ	− 80%	− 20%	− 28%	+ 109%	Alive
10	ICU	HFNC	ETT	HCQ	+ 41%	+ 412%	+ 33%	− 30%	Alive

Of note, protocol change (10 cc hAF I.V.) for patients 5–10. *N/A* not available, *ECMO* extracorporeal membrane oxygenation, *ETT* endotracheal intubation, *HFNC* high flow nasal cannula, *ICU* intensive care unit, *MACE* major adverse cardiac event, *NC* nasal cannula, *O2* oxygen, *Trach* tracheostomy, *Co-Rx* Co-treatment, *HCQ* hydroxychloroquine, *AZ* azithromycin; *Rem* remdesivir. Biomarker are represented as a percent change between study enrollment and termination of hAF (5 days) with focus on the last 6 patients under current protocol. CRP: C-reactive protein; IL-6: interleukin-6; LDH: lactate dehydrogenase

Biomarkers were evaluated before and after treatment with hAF (Table 1, Additional file 1: Table S1). Because of the small sample size and some missing data, it is difficult to make any broad statements concerning the influence of hAF on these biomarkers. That stated, in the latter cohort that received 10 cc hAF intravenously, a mean reduction in C-reactive protein by 38% was observed. Particularly notable is an apparent decrease in markers in those patients (4/6) that had improved clinical profiles. Conversely, two of the six patients saw increases in these inflammatory biomarkers—one had a prolonged hospitalization and the other was discharged to a rehabilitation hospital. One patient (#3) died on hospital day 8. She had multiple comorbidities including morbid obesity (BMI 55 kg/m^2^) and was extremely ill (maximal ventilatory support) on admission, and had a noticeable increase in her biomarkers. Outside of the above-mentioned issue related to aerosolized therapy, where theoretic concerns about safety to the provider delivering the nebulized hAF were never observed, there were no reported safety concerns.

## Discussion

The body of knowledge related to the pathophysiology and therapeutics of COVID is rapidly evolving. Our understanding of the relative contributions of innate and adaptive immune responses is similarly advancing. Indeed, many early observations have been modified as the nuances of different patient populations and presentations exist. Some patients, for example, demonstrate a syndrome consistent with cytokine storm, with a very aggressive inflammatory response. These patients might benefits from blockade of classic cytokine pathways (i.e. IL-6 antagonists). Other patients have a more subtle, perhaps less florid inflammatory response, and might not benefit as much from robust immune-modulation.

One of the attractive features of hAF is its diverse “soup” of ingredients. Expression profiling of 68 term gestation patients demonstrated that the cell-free AF transcriptome contains over 64,000 genes [5, 12]. At our center, we performed a cytokine array on AF from 17 patients with normal term pregnancy. Over 300 proteins (out of the 400 on the array) were present in AF with the majority associated with host defense and angiogenesis [12]. Biology has long taught us that blocking or augmenting a single pathway is confounded by naturally derived redundancy and alternative feedback loops. Indeed, while IL-6 is found to be upregulated in COVID patients, treatment with a singular antagonist does not necessarily change the course of the disease [17]. While some therapeutics will try to combine various mechanistic pathways[18], hAF is nature’s combination of not just one or two proteins, but rather thousands of proteins which have been mixed together and survived thousands of years.

Outside of the aforementioned studies surrounding wound healing, there is scant data with regards to the utilization of hAF for cardiopulmonary disease. Many COVID-19 patients, in addition to succumbing to acute hypoxic respiratory failure, are plagued with cardiac manifestations including myocarditis, accelerated heart failure, and arrhythmias [19]. Lung pathology specimens from the SARS-1 epidemic in 2005 demonstrated diffuse alveolar damage with extensive fibrosis [20]. Likewise, lung pathology in COVID-19 patients undergoing lung resection for lung cancer (i.e., non-autopsy pathology) revealed edema and patchy, inflammatory cellular infiltrates, particularly with macrophages [21]. Summarily, COVID-19 can cause an aggressive inflammatory and fibrotic response in both the heart and lung. This suggests that hAF, by mitigating inflammation and decreasing fibrosis, could impact the natural history of COVID-19 infected patients and provides the foundational platform for our investigation of the potential impact of systemic administration of hAF in COVID-19 patients.

## Limitations

This case series has a number of limitations including the small patient cohort (thereby limiting inferential and comparative statistics), mixed patient population including those in extremis, mixed delivery protocol between the first 4 and the last 6 patient, and concomitant medical and experimental therapies (for example, hydroxychloroquine—see Table 1). In addition, this case series was performed in the initial phases of the pandemic and some of the biomarker studies in the first 4 patients were not available or collected within the time frame set forth by protocol. Despite these significant caveats, the observations in these patients suggest that hAF can be used safely in this population and provides a potential signal of a favorable biologic influence. As such, this report provides some data to support the conceptual rationale for further investigation. This experience has propelled our application and subsequent approval from the FDA for IND status (#23,369), and has informed the design and recent implementation of a larger-scale, randomized clinical trial to evaluate the efficacy and novel utilization of human amniotic fluid as therapy for COVID-19.

## Supplementary Information

**Additional file 1: Table S1.** Raw biomarker data before and after therapy for each individual patient. N/A: not available; CRP: C-reactive protein; IL-6: interleukin-6; LDH: lactate dehydrogenase.

## Data Availability

The datasets generated and/or analyzed during the current study are not publically available due to protected health information but are available from the corresponding author on reasonable request.

## References

[1] Coronavirus 2019 (COVID-19): Pregnancy and breatfeeding [https://www.cdc.gov/coronavirus/2019-ncov/prepare/pregnancy-breastfeeding.html?CDC_AA_refVal=https%3A%2F%2Fwww.cdc.gov%2Fcoronavirus%2F2019-ncov%2Fspecific-groups%2Fpregnancy-faq.html]

[2] Chen H, Guo J, Wang C, Luo F, Yu X, Zhang W, Li J, Zhao D, Xu D, Gong Q (2020). Clinical characteristics and intrauterine vertical transmission potential of COVID-19 infection in nine pregnant women: a retrospective review of medical records. Lancet.

[3] Hamzelou J. Coronavirus: what we know so far about risks to pregnancy and babies. New Scientist 2020.

[4] Khan S, Jun L (2020). Nawsherwan, Siddique R, Li Y, Han G, Xue M, Nabi G, Liu J: Association of COVID-19 with pregnancy outcomes in health-care workers and general women. Clin Microbiol Infect.

[5] Tarca AL, Romero R, Pique-Regi R, Pacora P, Done B, Kacerovsky M, Bhatti G, Jaiman S, Hassan SS, Hsu CD (2020). Amniotic fluid cell-free transcriptome: a glimpse into fetal development and placental cellular dynamics during normal pregnancy. BMC Med Genomics.

[6] Cargnoni A, Di Marcello M, Campagnol M, Nassuato C, Albertini A, Parolini O (2009). Amniotic membrane patching promotes ischemic rat heart repair. Cell Transplant.

[7] Kim HG, Choi OH (2011). Neovascularization in a mouse model via stem cells derived from human fetal amniotic membranes. Heart Vessels.

[8] Mao Y, Pierce J, Singh-Varma A, Boyer M, Kohn J, Reems JA (2019). Processed human amniotic fluid retains its antibacterial activity. J Transl Med.

[9] Marsh KM, Ferng AS, Pilikian T, Desai AA, Avery R, Friedman M, Oliva I, Jokerst C, Schipper D, Khalpey Z (2017). Anti-inflammatory properties of amniotic membrane patch following pericardiectomy for constrictive pericarditis. J Cardiothorac Surg.

[10] O'Brien D, Kia C, Beebe R, Macken C, Bell R, Cote M, McCarthy M, Williams V, Mazzocca AD (2019). Evaluating the effects of platelet-rich plasma and amniotic viscous fluid on inflammatory markers in a human coculture model for osteoarthritis. Arthroscopy.

[11] Tahmasebi S, Tahamtan M, Tahamtan Y (2012). Prevention by rat amniotic fluid of adhesions after laparatomy in a rat model. Int J Surg.

[12] Pierce J, Jacobson P, Benedetti E, Peterson E, Phibbs J, Preslar A, Reems JA (2016). Collection and characterization of amniotic fluid from scheduled C-section deliveries. Cell Tissue Bank.

[13] Koob TJ, Rennert R, Zabek N, Massee M, Lim JJ, Temenoff JS, Li WW, Gurtner G (2013). Biological properties of dehydrated human amnion/chorion composite graft: implications for chronic wound healing. Int Wound J.

[14] Liu J, Sheha H, Fu Y, Liang L, Tseng SC (2010). Update on amniotic membrane transplantation. Expert Rev Ophthalmol.

[15] Mohammadi AA, Seyed Jafari SM, Kiasat M, Tavakkolian AR, Imani MT, Ayaz M, Tolide-ie HR (2013). Effect of fresh human amniotic membrane dressing on graft take in patients with chronic burn wounds compared with conventional methods. Burns.

[16] Tay MZ, Poh CM, Renia L, MacAry PA, Ng LFP (2020). The trinity of COVID-19: immunity, inflammation and intervention. Nat Rev Immunol.

[17] Stone JH, Frigault MJ, Serling-Boyd NJ, Fernandes AD, Harvey L, Foulkes AS, Horick NK, Healy BC, Shah R, Bensaci AM (2020). Efficacy of tocilizumab in patients hospitalized with Covid-19. N Engl J Med.

[18] Cavalcanti AB, Zampieri FG, Rosa RG, Azevedo LCP, Veiga VC, Avezum A, Damiani LP, Marcadenti A, Kawano-Dourado L, Lisboa T (2020). Hydroxychloroquine with or without azithromycin in mild-to-moderate Covid-19. N Engl J Med.

[19] Madjid M, Safavi-Naeini P, Solomon SD, Vardeny O (2020). Potential effects of coronaviruses on the cardiovascular system: a review. JAMA Cardiol.

[20] Tse GM, To KF, Chan PK, Lo AW, Ng KC, Wu A, Lee N, Wong HC, Mak SM, Chan KF (2004). Pulmonary pathological features in coronavirus associated severe acute respiratory syndrome (SARS). J Clin Pathol.

[21] Tian S, Hu W, Niu L, Liu H, Xu H, Xiao SY (2020). Pulmonary pathology of early-phase 2019 novel coronavirus (COVID-19) pneumonia in two patients with lung cancer. J Thorac Oncol.

