# Supplementary material for: A pilot trial of human amniotic fluid for the treatment of COVID-19
Source: BMC Res Notes. 2021 Jan 22;14:32. doi: 10.1186/s13104-021-05443-9 (PMC7820830; doi:10.1186/s13104-021-05443-9)
Supplement: Supplementary file 1 — Additional file 1: Table S1. Raw biomarker data before and after therapy for each individual patient. N/A: not available; CRP: C-reactive protein; IL-6: interleukin-6; LDH: lactate dehydrogenase. [file 13104_2021_5443_MOESM1_ESM.docx]

|  | **CRP**  (mg/dl) | | **IL-6**  (pg/ml) | | **D-dimer** (ug/ml) | | **LDH**  (U/L) | |
| --- | --- | --- | --- | --- | --- | --- | --- | --- |
| **Patient** | **Pre** | **Post** | **Pre** | **Post** | **Pre** | **Post** | **Pre** | **Post** |
| 1 | 23.1 | 21.4 | 57 | 248 | 2.1 | 14.3 | 530 | 387 |
| 2 | N/A | N/A | N/A | N/A | N/A | N/A | 313 | 317 |
| 3 | N/A | N/A | N/A | N/A | N/A | N/A | 439 | 336 |
| 4 | N/A | N/A | N/A | N/A | N/A | N/A | N/A | 249 |
| 5 | 8.2 | 1.3 | 10 | 4 | 1.9 | 1.5 | 395 | 307 |
| 6 | 3.7 | 0.3 | 4 | 4 | 1.9 | 1.1 | N/A | N/A |
| 7 | 15.6 | 22.8 | 22 | 46 | 1.6 | 14.7 | 539 | 679 |
| 8 | 7.8 | 1 | 4 | 4 | 1.3 | 1.3 | 400 | 315 |
| 9 | 12.4 | 5.9 | 5 | 4 | 0.7 | 0.6 | 241 | 261 |
| 10 | 20.4 | 28.8 | 65 | 333 | 2.7 | 3.6 | 551 | 384 |

**Table S1**. Raw biomarker data before and after therapy for each individual patient. N/A: not available; CRP: C-reactive protein; IL-6: interleukin-6; LDH: lactate dehydrogenase.
